# Catalytically Active Hollow Fiber Membranes with Enzyme‐Embedded Metal–Organic Framework Coating

**DOI:** 10.1002/anie.202003287

**Published:** 2020-06-25

**Authors:** Daniel Josef Bell, Monika Wiese, Ariel Augusto Schönberger, Matthias Wessling

**Affiliations:** ^1^ Chemical Process Engineering RWTH Aachen University Forckenbeckstr. 51 52074 Aachen Germany; ^2^ DWI Leibnitz-Institute for Interactive Materials Forckenbeckstr. 50 52074 Aachen Germany

**Keywords:** enzymes, membranes, metal–organic frameworks, oxidation, polymers

## Abstract

Metal–organic frameworks (MOFs) are suitable enzyme immobilization matrices. Reported here is the in situ biomineralization of glucose oxidase (GOD) into MOF crystals (ZIF‐8) by interfacial crystallization. This method is effective for the selective coating of porous polyethersulfone microfiltration hollow fibers on the shell side in a straightforward one‐step process. MOF layers with a thickness of 8 μm were synthesized, and fluorescence microscopy and a colorimetric protein assay revealed the successful inclusion of GOD into the ZIF‐8 layer with an enzyme concentration of 29±3 μg cm^−2^. Enzymatic activity tests revealed that 50 % of the enzyme activity is preserved. Continuous enzymatic reactions, by the permeation of β‐d‐glucose through the GOD@ZIF‐8 membranes, showed a 50 % increased activity compared to batch experiments, emphasizing the importance of the convective transport of educts and products to and from the enzymatic active centers.

## Graphical abstract

**Passing through**: Reported for the first time is the successful in situ biomineralization of an enzyme‐containing metal–organic framework (MOF) layer on a polymeric hollow fiber in a facile one‐step process. The interconnection between an enzymatic active layer and a porous membrane overcomes stability drawbacks, removes the need for MOF separation, and leads to increased enzyme activity as a result of the improved mass transport by permeation.

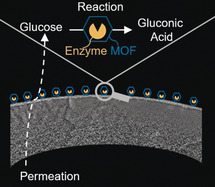

## Introduction

Enzymatic reactions enable selective reactions under mild reaction conditions such as low temperatures and aqueous environments, making them a promising alternative for more environmentally friendly synthesis in the (bio)chemical industry.^1^ However, the limited enzymatic stability, the difficult separability of the product, and the enzyme recycling pose major challenges. To make a biotechnological process more favorable, these challenges must be addressed.

Enzyme immobilization either on or in different carrier materials is a promising method to overcome these challenges.^2^ Metal–organic frameworks (MOFs), which are three‐dimensional nanoporous materials consisting of inorganic nodes and organic linkers, possess special properties that make them predestined for enzyme immobilization.^3^, ^4^, ^5^ These include a precisely adjustable three‐dimensional structure and a defined chemical microenvironment that counteracts enzyme denaturation.^6^ Various MOFs have so far been successfully used for enzyme immobilization. The immobilization can be performed by either post‐synthetic adsorption/infiltration methods or in a single step during MOF synthesis.^7^, ^8^, ^9^, ^10^, ^11^, ^12^ In situ biomineralization is a particularly interesting single‐step method as it allows the immobilization of enzymes inside the MOF structure independent of the relationship between enzyme and pore size.^6^, ^13^, ^14^, ^15^ The concept of in situ biomimetic mineralization is inspired by the natural process of biomineralization, which builds defined molecular architectures by self‐assembly processes between organic and inorganic building blocks, forming hierarchically structured materials.^16^, ^17^ Different biomacromolecules like polysaccharides, proteins, DNA, and living cells have been successfully incorporated into MOF structures by biomimetic mineralization.^7^, ^13^, ^14^, ^18^, ^19^, ^20^ It was demonstrated that biomolecules can act as a seed for the mineralization process, depending on its surface properties, as the MOF building blocks accumulate around these biomolecules by intermolecular hydrogen bonding, and electrostatic and hydrophobic interactions, leading to crystal growth around the biomolecule.^13^, ^21^ The most studied MOF for this approach is the zeolite imidazolate framework‐8 (ZIF‐8) since it is porous, stable in most solvents, and can be synthesized in an aqueous media at ambient temperature and pressure.^6^ These mild synthesis conditions are a major advantage because the sensitive biomolecules need to be present within the reaction mixture during MOF synthesis, and denaturation should be prevented.^4^

A publication from 2015 describes the first successful in situ biocrystallization in ZIF‐8 crystals in a water‐based system.^13^ It has been shown that enzyme activity and stability to harsh reaction conditions is improved by the immobilization process. Liang et al. showed that the enzyme HRP has an activity of 88–90 % after exposure to trypsin or boiling in water and DMF.^13^ However, the recovery of the finely distributed MOF crystals after the enzymatic reaction poses a major challenge.^20^

(Bio)catalytic active membranes, which are conventional membranes functionalized with (bio)catalysts, combine the catalytic reactions and the separation of reactants and products in one step. Given the immobilization of the (bio)catalysts onto the membranes, catalyst recycling is possible.^22^, ^23^, ^24^, ^25^, ^26^, ^27^ We envision the immobilization of enzyme‐active ZIF‐8 MOFs onto commercial hollow fiber membranes, combining the benefits of biocatalytic membranes and enzymatic‐active MOFs, allowing continuous enzymatic reaction without the need of MOF separation from the products. In recent years, polymeric membranes have been successfully coated with pure MOF layers, mainly by four different processes. These processes include direct synthesis by one‐step in situ growth, stepwise film growth, seeded growth, and counter‐diffusion synthesis.^28^, ^29^, ^30^ These synthesis strategies, including in situ growth, seeded growth and counter‐diffusion, made it possible to produce defect‐free, uniform layers that are strongly intergrown with the membrane.^31^, ^32^, ^33^ These membranes have so far been successfully used for adsorbents and gas separation tasks.^29^, ^34^ Typical applications are the separation of light gases like H_2_, CO_2_, N_2_, CH_4_, and mixtures of hydrocarbons like C_3_H_6_/C_3_H_8_ or CH_2_H_4_/C_2_H_6_.^33^, ^35^, ^36^, ^37^, ^38^, ^39^, ^40^, ^41^, ^42^, ^43^, ^44^ These separations require defect‐free layers to prevent nonselective convection and only allow molecularly selective diffusion. In fact, for the conversion addressed here, diffusive transport is too slow, but convection is desired.

However, so far, only two working groups have successfully modified a membrane with an enzyme‐containing MOF layer. Zhang et al. deposited preformed ZIF‐8 crystals, which were loaded with the enzyme carbonic anhydrase by adsorption on a modified flat sheet PAN membrane.^45^ These MOF crystals serve as seeds for the crystallization of the subsequent in situ growth of a defect‐free ZIF‐8 layer. The membrane showed an improved CO_2_/N_2_ selectivity of 9 to 165.5 compared to the enzyme‐free membrane. A further publication from 2018 describes the modification of PAN ultrafiltration membranes in a complex multistage process with polyethyleneimines (PEIs), MOFs, lactase, and polydopamine for the removal of micropollutants from wastewater.^46^ However, this process does not produce interconnected MOF layers. Both of so far described concepts rely on adsorptive enzyme binding to preformed MOF crystals, which can cause enzyme leaching and utilize time‐consuming multistep synthesis methods.^46^ Furthermore, these publications do not demonstrate the modification of hollow fiber membranes, which are favorable since they allow an easier module design and a higher packing density. The coating of organic and inorganic hollow fibers with different MOF's like HKUST‐1, ZIF‐8, ZIF‐93, MOF‐74, and UTSA‐16 is already described in the literature.^44^, ^47^, ^48^, ^49^, ^50^, ^51^ Direct crystallization methods usually need harsh reaction conditions like elevated temperatures and microwave irradiation to enhance the heterogeneous nucleation.^30^ The application of seed crystals or surface functionalization poses a way to enhance the heterogeneous nucleation. However, these processes are time‐consuming and hard to scale‐up. Furthermore, this method produces a large amount of waste because of bulk MOF formation.^15^, ^52^ Counter‐diffusion synthesis poses a relatively new method to synthesize MOF's on polymeric membranes avoiding bulk MOF formation.^15^, ^52^ Synthesis at the interface between an organic and aqueous phase is a promising way for membrane synthesis since it restricts the MOF formation to a confined space and leads to dense MOF layers interconnected with the membrane without the need for surface functionalization.^47^, ^51^, ^53^, ^54^

**Figure 1:**
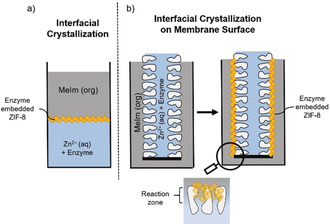
a) Schematic representation of the in situ biomineralization of enzyme embedded MOFs by interfacial crystallization at a liquid–liquid interface. b) Schematic representation of the interfacial crystallization of enzyme embedded MOFS within the reaction zone at the hollow fiber membrane shell‐side.

Herein, we report for the first time the in situ biomimetic mineralization of enzyme‐containing ZIF‐8 layers on a polymeric hollow fiber membrane [Polyethersulfone (PES)] by a single‐step counter‐diffusion process with two immiscible phases, leading to ZIF‐8 formation within a reaction zone located at the membrane shell side (Figure 1). The described counter‐diffusion process is inspired by the interfacial polymerization (IP) method, which is the established industrial manufacturing process for reverse osmosis membranes. During the interfacial polymerization, two different monomers dissolved in either the organic or the aqueous phase, polymerize at the interface between two immiscible liquids.^55^ In this publication, the two monomers are substituted by the inorganic nodes and the organic linker, leading to an interfacial crystallization. Interfacial polymerization has proven to be suitable for polymerizing a thin enzymatic active pepsin layer onto a synthetic porous membrane.^23^ These enzymatically active ultrathin pepsin membranes digest proteins and pass the products selectively once their retention is low enough. This process is represented by the slow kinetics in the order of hours. It would also be desirable to have access to enzymatically active membranes where single passage and short residence times of seconds results in the desired chemical conversion. Counter‐diffusion processes have been shown to produce defect‐free, intergrown MOF films with controllable thickness without the need for further surface modification.^54^ Most publications describe the counter diffusion process for inorganic membrane materials. Only a few publications describe the successful utilization of this method for the coating of polymeric flat sheet membranes^54^, ^56^, ^57^ or even hollow fiber membranes.^47^, ^51^ However, this method has not been tested for the in situ biomineralization of enzymes. Besides the proof of concept for this method to produce enzymatic active MOF layers, the transition from batch experiments to continuous enzymatic reaction shall be evaluated. For this purpose, the reaction mixture is permeated with different flowrates trough the coated hollow fiber membranes and the product concentration is monitored at the permeate outlet to evaluate the coating stability and the influence of the convective flow on the enzymatic activity.

## Results and Discussion

### Interfacial Crystallization of GOD@ZIF‐8

**Figure 2:**
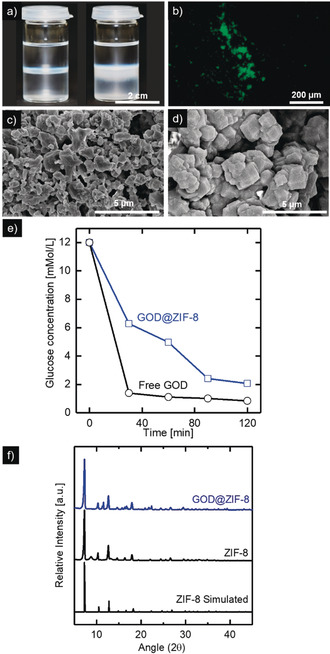
a) In situ biomineralization of GOD@ZIF‐8 at the liquid–liquid interface between an aqueous Zn(CH_3_COO)_2_ and an organic (Hexane + MeOH + EtOH) MeIm solution. b) Fluorescence microscopy image of GOD@ZIF‐8 crystals synthesized with NCS‐fluorescein labeled GOD. c) SEM images of ZIF‐8‐ layers without added GOD. d) SEM images of ZIF‐8‐ films with 2 mg mL^−1^ GOD in the aqueous phase. e) Enzymatic activity test of GOD@ZIF‐8 crystals and free GOD in PBS buffer with 12 mm glucose. f) XRD pattern of synthesized pure ZIF‐8 samples and ZIF‐8 samples with embedded GOD.

Counter diffusion methods are well suited for the synthesis of MOF layers at the interface between two liquids with limited miscibility.^58^, ^59^, ^60^ In recent publications, this concept shows promising results for the coating of porous membrane structures.^54^, ^61^ In this publication, the interfacial synthesis of ZIF‐8 crystals with embedded glucose oxidase (GOD) should be performed at the interface between an organic (hexane + methanol + ethanol) 2‐methylimidazole and an aqueous zinc acetate [Zn(CH_3_COO)_2_] + GOD solution. The flavoprotein glucose oxidase catalyzes the oxidation of β‐d‐Glucose to d‐gluconolactate and is a common model enzyme because of its high stability.^62^, ^63^ To evaluate the suitability of this method for the enzyme in situ biomineralization concerning crystal morphology, enzyme inclusion, and enzymatic activity, the interfacial synthesis was performed in 3 mL vials (Figure 2 a). Immediately after overlaying the aqueous with the organic phase, the interface becomes turbid (Figure 2 a, left), showing the spontaneous formation of ZIF‐8 crystals at the interface. This spontaneous formation of a white precipitate during either conventional or interfacial ZIF‐8 synthesis was also reported in the literature.^19^, ^54^ During 3 hours of reaction, more ZIF‐8 is formed at the interface and partially starts to sediment (Figure 2 a, left). Figures 2 c and d show the SEM images of the formed ZIF‐8 and GOD@ZIF‐8 crystals after three hours of reaction time. The SEM image of the pure ZIF‐8 (Figure 2 c) shows a well‐intergrown layer composed of small (<1 μm) ZIF‐8 crystals. In contrast to this, the morphology of the GOD@ZIF‐8 layer (Figure 2 d) shows larger (1–3 μm) and more regular ZIF‐8 crystals. A gradient test with different GOD concentrations between 0 mg mL^−1^ and 4 mg mL^−1^ (see Figure S3 in the Supporting Information) shows the formation of larger and more homogenous crystals in the presence of GOD. The formation of larger crystals in the presence of proteins is also reported in literature and might be attributed to a local enrichment of metal ions and linkers around the proteins leading to facilitated crystallization of ZIF‐8 around the proteins.^13^ The temporal evolution of the particles within a 4 hour reaction time is presented in Figure S2. The results show that in the beginning, small ZIF‐8 crystals form, which grow as the reaction progresses. After 3 hours of reaction time, the crystal growth slows down, indicating that the reaction is complete. In addition, XDR analysis of the obtained MOF powders shows the characteristic peaks for ZIF‐8 (Figure 2 f). Fluorescence microscopy images of the formed ZIF‐8 crystals (Figure 2 b) show an evenly distributed fluorescence of the ZIF‐8 crystals revealing the successful enzyme immobilization. Confocal laser scanning microscopy (see Figure S7) also revealed a homogenous enzyme distribution within the ZIF‐8 crystals, highlighting that the enzymes are encapsulated and not only adsorbed onto the surface. This finding is in agreement with literature on GOD@ZIF‐8 crystals.^64^, ^65^ The encapsulation can be explained by the enzyme affinity towards the Zn^2+^ ions and the organic linker, leading to prenucleation around the enzymes.^13^ In addition to labeling experiments, the enzyme encapsulation efficiency was determined according to Equation (S1) (see the Supporting Information) by a colorimetric bicinchoninic acid (BCA) protein assay. During the interfacial crystallization, the encapsulation efficiency is 25 %. Figure 2 e shows the enzymatic activity test of the synthesized GOD@ZIF‐8 crystals and the free enzyme performed in PBS buffer with 12 mm glucose at 37 °C. The glucose concentration decreases from 12 to 6 mm within the first 30 minutes and reaches nearly full conversion (2 mm) of glucose after 2 hours, showing the successful immobilization of active enzyme. The steady but not linear glucose conversion can be explained by the decrease in substrate concentration. The immobilized enzyme shows reduced enzymatic activity in comparison to the free enzyme. This decreased activity may be caused by the restricted transport of products and educts to the active centers or a partial denaturation during the immobilization procedure.

### Membrane Coating with GOD@ZIF‐8

**Figure 3:**
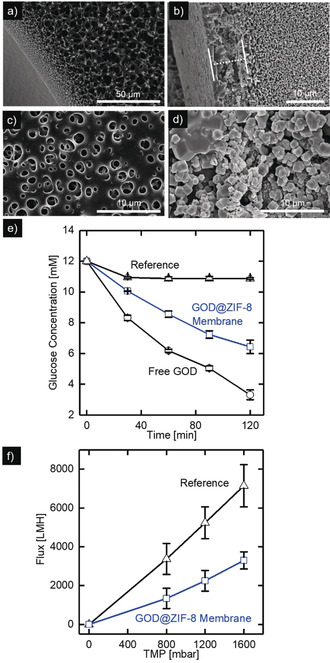
a,c) SEM images of an uncoated PES hollow fiber membrane and b,d) a PES hollow fiber membrane coated with a GOD@ZIF‐8 layer by interfacial crystallization. e) Enzymatic activity of a GOD@ZIF‐8 coated membrane. f) Pure water flux measurement performed with DI water under constant pressure conditions in dead‐end mode during inside out permeation for an uncoated (black) and GOD @ ZIF coated membrane (blue). All error bars represent the standard deviation based on three independent measurements of three different membranes.

The promising concept of GOD@ZIF‐8 crystallization at a liquid–liquid interface is applied as a coating process to generate an enzyme‐containing ZIF‐8 layer on top of commercially available PES 200 polymeric hollow fiber membranes. For the membrane coating step, the membrane pores and the membrane lumen is filled with a GOD containing aqueous Zn(CH_3_COO)_2_ solution and subsequently placed into the organic phase (hexane + methanol 1.9 vol. % + ethanol 2.5 vol. %) containing the organic linker 2‐methylimidazole. After a reaction time of 3 hours at 20 °C, GOD@ZIF‐8 functionalized membranes are dried to evaporate the hexane and cleaned with ultrapure water. The coating morphology was investigated by scanning electron microscopy. Figure 3 shows the respective SEM images of the uncoated (a, c) and the GOD@ZIF‐8 coated membrane (b, d).

The cross‐section images (Figure 3 a,b) show the successful deposition of a crystalline MOF layer on the membrane shell side, whereas no MOF layer is present in the membrane lumen (see Figure S4). Recent publications have shown that the localization of the Zn^2+^ solution is predominantly responsible for the layer location.^47^, ^51^ In our case, the hydrophilic support membrane, which is filled and prewetted with aqueous Zn^2+^ solution leads to a localization of the reaction zone directly on the shell side. The first ZIF‐9 crystals act as a barrier for the permeation of the organic phase into the membrane, restricting the ZIF‐8 growth to the shell side. These images prove the suitability of the counter diffusion process for the in situ biomineralization of GOD onto a hollow fiber surface. The layer has a thickness of 8 μm and consists of intergrown large MOF crystals with a size between 1–3 μm. The MOF layer penetrates to some extent into the porous membrane matrix indicating a layer growth predominantly from the organic to the aqueous phase wetting the polymer support. In the literature, enzyme‐free MOF layers with a thickness of 8.8 micrometers and 10–25 μm were synthesized on polymeric membranes by the counter diffusion concept.^47^, ^51^ The top‐view SEM images (Figure 3 c,d) show a compact GOD@ZIF‐8 layer composed of small intergrown crystals, covering the entire membrane surface. However, the GOD@ZIF‐8 layer has some defects and is not entirely dense. These defects can be attributed to the limited number of Zn^2+^ ions under static coating conditions and the presence of the enzyme, which concentrates the metal ions in their surrounding acting as nucleation centers and thereby affecting the growth process.^13^, ^51^, ^56^

To evaluate the biomineralization of GOD inside the MOF coating, the protein content inside the MOF layer is analyzed by a colorimetric BCA protein assay in a triple determination. The measured protein content on the membrane was 29±3 μg cm^−2^, showing the successful biomineralization.

The enzymatic activity of the GOD@ZIF‐8 coated membranes was analyzed by incubation of coated membrane pieces [3 cm (1.89 cm^2^)] in a 12 mm glucose solution at 37 °C. The decrease in glucose concentration is measured at regular time intervals. Figure 3 e shows the corresponding data. Each data point is the average value of three independent measurements. To identify the glucose conversion caused by adsorbed enzymes on the membrane surface, a bare PES was exposed to the same coating and cleaning procedure but without the addition of the organic crosslinker during the in situ biomineralization. This reference membrane leads to an initial decrease in glucose concentration from 12 to 11 mm, which can be attributed to dilution effects caused by absorbed water in the membrane. After this initial decrease, the reference membrane shows no enzymatic activity, indicating that GOD does not significantly adsorb on the membrane surface since both have a negative surface charge at a neutral pH.^62^, ^66^

In contrast, the GOD@ZIF‐8 coated membrane shows a continuously decreasing glucose concentration. Within two hours, 5.5 mm glucose is converted and the concentration decreases nearly linear over time. This data proves the successful immobilization of active GOD within the MOF layer on the membrane shell side.

Additionally, the small error bars demonstrate a reproducible membrane coating method. Reference experiments with the free enzyme show the activity loss caused by the immobilization. For this purpose, the glucose conversion by 50 μg free GOD, which is the measured amount of enzyme in the ZIF‐8 coating, is monitored. The free enzyme shows higher activity and converts 9 mm glucose in two hours. The decreased activity of the immobilized enzyme can be caused by mass transport limitations or by changes in the enzyme structure. However, the results demonstrate the successful coating of a commercial polymeric hollow fiber with an enzymatic active MOF layer.

### Continuous Enzymatic Reaction Inside a Membrane Module

To evaluate the influence of the GOD@ZIF‐8 coating on the membrane performance, the pure water permeability of a coated GOD@ZIF‐8 membrane and a reference membrane was investigated. Figure 3 f depicts the results of the pure water flux measurements for an uncoated PES 200 reference membrane and a coated PES 200 membrane. All experiments were performed in dead‐end mode by inside out permeation. Each data point represents the mean value of three independent measurements, while the respective error bars depict the standard deviation. For the reference membrane, the error bar is in the range of 15 %. The coated membrane shows similar error bars indicating a reproducible coating method. For both membranes, the flux increases linear with increasing transmembrane pressure (TMP), indicating a stable layer. If the layer would not stable during inside out permeation, parts of the coating can delaminate from the membrane, changing the membrane resistance and leading to a nonlinear flux increase. In comparison to the uncoated membrane, the permeability of the coated membrane decreases by 54 %. The reason for the decreased permeability is the additional transport resistance resulting from the ZIF‐8 layer and partial pore blocking.

However, the results show that the MOF layer is not entirely dense, allowing the permeation of water, which is an essential prerequisite for a continuous enzymatic reaction in a liquid phase.

**Figure 4:**
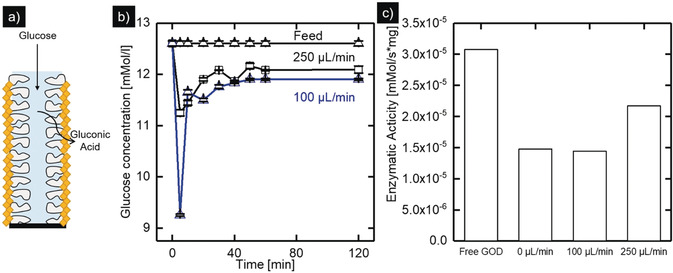
a) Schematic representation of the continuous enzymatic conversion of glucose on a GOD @ ZIF 8 membrane. b) Concentration profiles of glucose for different flowrates. c) Comparison of the calculated conversion rates.

For the investigation of the continuous enzymatic conversion of glucose, coated membranes (85 mm membrane length) were mounted into tubular dead‐end modules with a total module volume of 3.4 mL (see Figure S3). Fresh glucose solution with a concentration of 12.6 mm L^−1^ is pumped at a constant flow rate into the membrane lumen channel, permeating through the membrane wall, and passing the enzymatic active GOD@ZIF‐8 layer where glucose is converted to gluconic acid (see Figure 4 a). The glucose concentration at the module outlet was measured for different flow rates over a time period of 120 minutes after the module is filled with glucose solution. Figure 4 b shows the resulting concentration profiles of the feed and the permeate for a feed flow rate of 250 μL min^−1^ and 100 μL min^−1^. For both flowrates, an increased glucose conversion is detected in the first 5 minutes. This low glucose concentration can be caused by initial dilution of the feed solution by water adsorbed in the membrane porosity. After this initial high glucose conversion, the permeate concentration approaches a constant value for the duration of the experiment. For a flowrate of 250 μL min^−1^ (residence time of ≈816 s), a permeate concentration of 12.1 mm L^1^ is reached. For a decreased flow rate of 100 μL min^−1^ (residence time of ≈2040 s), the permeate glucose concentration decreases to 11.9 mm L^1^, which can be explained by the longer residence time. This constant glucose conversion over the entire experiment time shows that no significant enzyme leaching occurs, highlighting the coating stability for operation under continuous flow.

Since the transport of educts and products to the active enzyme sites is expected to have a significant impact on the enzymatic conversion rate, the experiments performed under constant flow conditions are expected to show an increased conversion rate in comparison to experiments without flow. To determine the influence of mass transport limitations on the enzymatic conversion of glucose, the conversion rates of the batch and the continuous process were compared. To achieve the comparability of these two processes, the conversion rate in the batch process was determined, taking into account the concentration change from Figure 3 e during the first 30 minutes (assuming linear degradation), the volume of the glucose solution, and the enzyme mass according to Equation (1). For the determination of the conversion rate for the continuous process, we analyzed the concentration difference between feed and permeate (Δ*C*_Glucose_), the residence time in the module (*τ*), the module volume (*V*_Module)_ and the enzyme mass (*m*_GOD_) according to Equation (2).

(1) $$\frac{\Delta C_{Glucose}V_{Solution}}{\Delta tm_{GOD}}=conversion\;rate\left[\frac{mMol}{\mu g•s}\right]$$

(2) $$\frac{\Delta C_{Gucose}V_{Module}\;\;\;}{\tau m_{GOD}\;}=conversion\;rate\left[\frac{mMol}{\mu g•s}\right]$$

Figure 4 c depicts the calculated conversion rates of the free enzyme, the batch, and the continuous reaction. The free enzyme serves as a benchmark to determine the influence of immobilization on enzyme activity. The free enzyme shows the highest conversion rate of 3.1×10^−5^ mm s^−1^ mg and a turnover frequency (TOF) of 4.9 s^−1^. The enzyme immobilized on the membrane shows a reduced enzymatic activity by 50 % (TOF 2.4 s^−1^) in the batch experiment. Diffusion limitation resulting from the encapsulation or a partial change in the enzyme structure during the immobilization procedure can cause a decrease in enzyme activity. Convective transport of educts to the enzymatic active centers by permeation poses a promising way to overcome the diffusion limitation. During the continuous enzymatic reaction in the membrane module at a low flow rate of 100 μL min^−1^, an enzymatic activity corresponding to the batch experiment is reached. When the flow rate is increased to 250 μL min^−1^, the enzymatic activity increases by a factor of 50 % to 2.2×10^−5^ mm s^−1^ mg (TOF 3.5 s^−1^) compared to the batch reaction. This improved enzymatic activity emphasizes the importance of the convective transport of educts and products to the enzymatic active centers. These results demonstrate the suitability of GOD@ZIF‐8 membranes for continuous enzymatic reactions showing high stability and improved enzymatic activity.

## Conclusion

In the present study, we presented the coating of a PES hollow fiber membrane with an enzyme‐embedded ZIF‐8 layer by a single‐step interfacial biomineralization method. The technique yielded well, adhering uniform and intergrown ZIF‐8 layers on the membrane shell side. Given its simplicity, the technique enables the large‐scale production of coated membranes. Enzyme quantification measurements and activity tests revealed the successful immobilization of active GOD. For the first time, polymeric hollow fiber membranes are successfully coated with permeable enzyme embedded MOF layers by an interfacial biomineralization method. The coated membranes represent an enzyme membrane reactor capable of converting the substrate during permeation. We could show that the convective transport of educts and products during permeation increases the enzymatic activity by 50 % when applying high feed flow rates. During the experiments, no loss in activity over time was detectable, highlighting the coating stability and emphasizing the applicability of these composite membranes for continuous enzymatic reactions. The work highlights the potential of interfacial crystallization of enzyme embedded MOFs onto porous synthetic membranes for efficient membrane bioreactors. The presented method can be transferred to other enzymes and membranes as long as the membrane is permeable for the enzyme and the MOF precursors.

## Conflict of interest

The authors declare no conflict of interest.

## Supporting information

As a service to our authors and readers, this journal provides supporting information supplied by the authors. Such materials are peer reviewed and may be re‐organized for online delivery, but are not copy‐edited or typeset. Technical support issues arising from supporting information (other than missing files) should be addressed to the authors.

Supplementary
