# Supplementary material for: Catalytically Active Hollow Fiber Membranes with Enzyme‐Embedded Metal–Organic Framework Coating
Source: Angew Chem Int Ed Engl. 2020 Jun 25;59(37):16047–53. doi: 10.1002/anie.202003287 (PMC7540569; doi:10.1002/anie.202003287)
Supplement: Supplementary file 1 — Supplementary [file ANIE-59-16047-s001.pdf]

Angewandte Chemie
Eine Zeitschrift der Gesellschaft Deutscher Chemiker

Supporting Information

Catalytically Active Hollow Fiber Membranes with Enzyme-Embedded Metal–Organic Framework Coating

*Daniel Josef Bell, Monika Wiese, Ariel Augusto Schönberger, and Matthias Wessling**

anie_202003287_sm_miscellaneous_information.pdf

Supporting Information

Additional Measurements and Experimental Details

Materials

The listed chemicals are used without further purification: , 2-methylimidazole (99%, Sigma-Aldrich), D-(+)-glucose (99.5%, Sigma-Aldrich), di-sodium hydrogen phosphatedihydrate (99%, Merck), 3,5-dinitrosalicylic acid (98%, Merck), ethanol (99.5%, VWR), n-hexane (99%, VWR), methanol (99.9% Sigma-Aldrich), 2-propanol (technical, VWR), potassium chloride (99%, VWR), potassium dihydrogen phosphate (99%, Carl Roth), potassium sodium tartrate tetrahydrate (99%, Merck), sodiumchloride (99.8%, VWR), sodium hydroxide (99%, Merck), hydrochloric acid (32%, Carl Roth), phosphate buffered saline tablets (Sigma-Aldrich), NHS-Fluorescein (Thermo Fischer) and zinc acetate dihydrate (99%, Sigma-Aldrich). Glucose oxidase (GOD) from Aspergillus niger (VWR) is applied for enzyme@MOF experiments. Protein quantification is performed with a BCA protein assay kit from Alfa Aesar.

Interfacial biomineralization of free-standing ZIF-8 and GOD@ZIF-8 films

The interfacial synthesis of ZIF8 and GOD@ZIF8 crystals is performed in glass vials. In a typical experiment, 3 mL aqueous zinc acetate solution (85 mM/l) and 3 mL of the organic 2-methylimidazole solution 66 mM/L were layered on top with a pipette. In the case of GOD@ZIF8 synthesis, GOD is added to the aqueous solution to a concentration of 2 mg/ml. After 3 hours reaction time at room temperature, the liquid phases were removed and the solid powder was washed twice with 5 mL isopropanol via resuspending and centrifugation. The obtained solid was dried at room temperature overnight and stored at 4°C.

WILEY-VCH

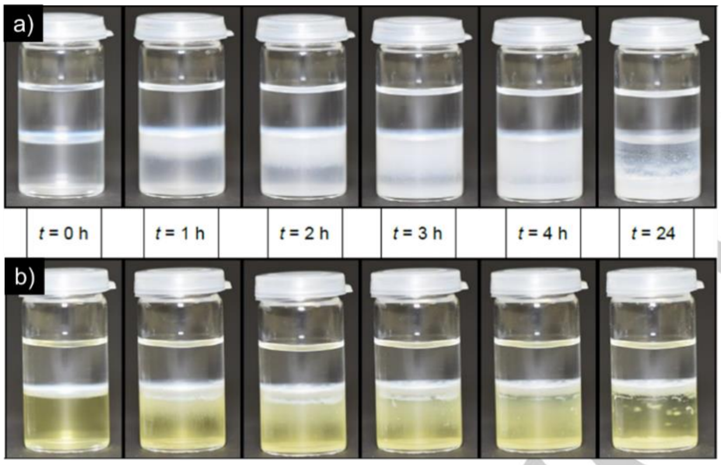

Figure S1. Time evolution of ZIF-8 crystals at the phase boundaries between an aqueous MeIm and an organic (Hexane + MeOH + EtOH) ZnAc phase (a) without the addition of GOD (b) with 2 mg/ml GOD in the aqueous phase.

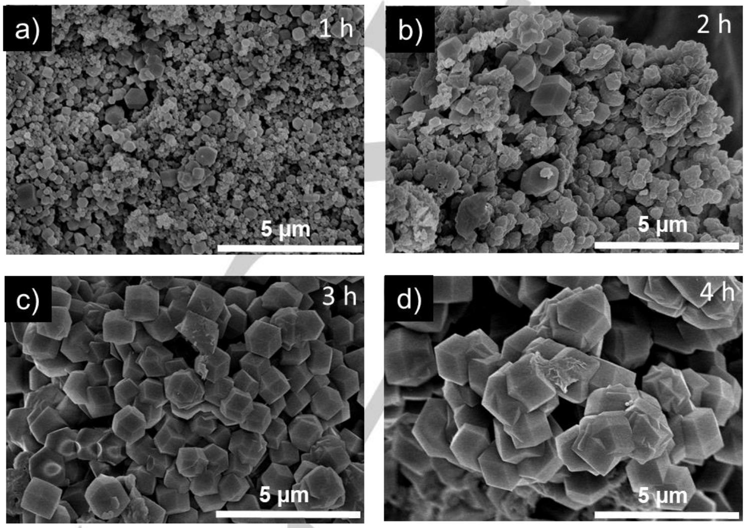

Figure S2. Time evolution of ZIF-8 crystals synthesized at the phase boundaries between an aqueous MeIm and an organic (Hexane + MeOH + EtOH) ZnAc phase with 2 mg/ml GOD in the aqueous phase, (a) 1 h reaction time, (b) 2 h reaction time, (c) 3 h reaction time, (d) 4 h reaction time.

WILEY-VCH

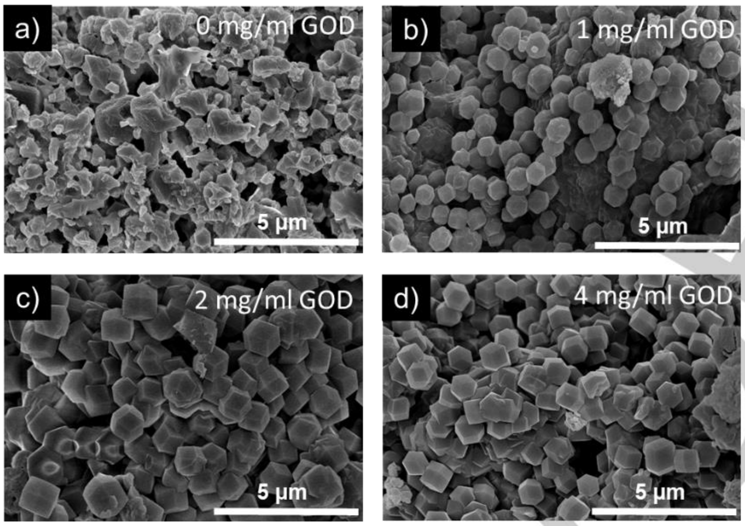

Figure S3. ZIF-8 crystals synthesized at the phase boundaries between an aqueous MeIm and an organic (Hexane + MeOH + EtOH) ZnAc phase with different GOD concentration in the aqueous phase, (a) 0 mg/ml GOD, (b) 1 mg/ml GOD, (c) 2 mg/ml GOD, (d) 3 mg/ml GOD.

Fabrication of GOD@ZIF-8 films on hollow fiber membranes

The coating of commercially available polyethersulfone (PES) hollow fiber membranes with an average pore diameter of 200 nm (Pentair X-Flow MF 02, 2.2mm outer diameter, 1.5mm inner diameter) was performed using the same interfacial synthesis method based on an aqueous GOD containing zinc acetate and an organic 2-methylimidazole solution. For the membrane coating step, 10 cm long hollow fibers were sealed at the bottom and connected to a peristaltic pump. Before the actual coating step, the membranes were extensively cleaned via inside out filtration of a water-ethanol mixture and ultrapure water, each for 30 minutes at a constant flux of 35 LMH. After the cleaning step, the aqueous zinc acetate solution is filtered inside out through the membrane at a constant flux of 35 LMH for 30 minutes. The zinc acetate filled membrane is placed into the organic solution to induce the interfacial reaction. After a reaction time of 3 hours, the closed membrane end is cut off to remove the liquid phase from the membrane lumen, and then the membrane is dried in air to evaporate the hexane. In the last step ultrapure water is filtered through the membrane at 35 LMH for 30 minutes and the membranes are stored at 4°C until further. experiments.This cleaning step is essential to remove loosely adsorbed enzymes from the membrane and the ZIF-8 surface (see Figure S5)

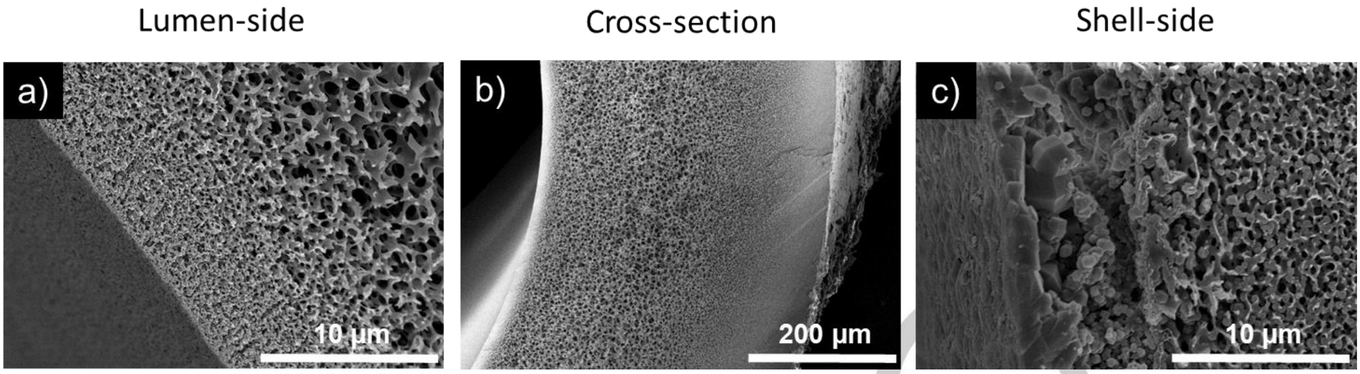

Figure S4. GOD@ZIF-8 coated PES hollow fiber membrane. High-resolution image of the membrane lumen (a), overview cross-section image (b), high-resolution image of the membrane shell (c).

Enzymatic activity of GOD@ZIF-8 and GOD@ZIF-8 membranes

The enzymatic activity of GOD@ZIF8 and GOD@ZIF8 coated membranes was tested by measuring the glucose conversion in PBS buffer at pH 7.4 with an initial glucose concentration of 12 mM. For the analysis of GOD@ZIF8 crystals prepared at the liquid-liquid interface, the entire obtained solid was suspended in 1850 µL PBS buffer and incubated on a thermoshaker at 37°C. The enzymatic activity of GOD@ZIF8 coated membranes is measured by immersing three 1 cm long membrane pieces into 850 µL PBS buffer at 37°C. Samples of 100 µL were taken for glucose quantification at regular time intervals. The samples were heated to 90°C for 15 minutes to stop the enzymatic reaction and stored at -21°C until further analysis.

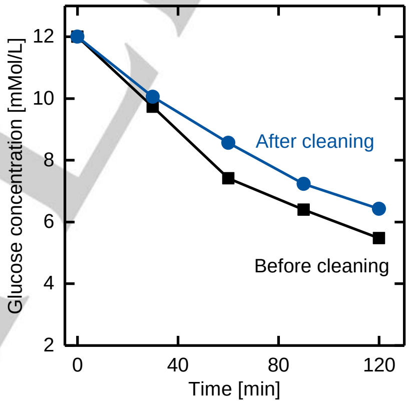

Figure S5. Time evolution of glucose concentration during enzymatic conversion with GOD@ZIF-8 membranes. The data points represent the enzymatic conversion by a membrane directly after the coating procedure without the subsequent cleaning step (black) and with the following cleaning step (blue).

Enzymatic activity of GOD@ZIF-8 membranes during permeation

GOD@ZIF8 coated membranes with a length of 10 cm are mounted into tumular dead-end modules, as shown in Figure S6, leading to an effective membrane length of 8.5 cm. The membrane module has a total volume of 3.4 mL. For the continuous enzymatic reaction, the whole module is immersed into a water bath at 37°C, and the glucose solution permeates from the lumen side through the membrane at different flow rates by a syringe pump. Samples for the glucose quantification are taken at regular time intervals at the module outlet. The samples were heated to 90°C for 15 minutes to stop the enzymatic reaction and stored at -21°C until further analysis.

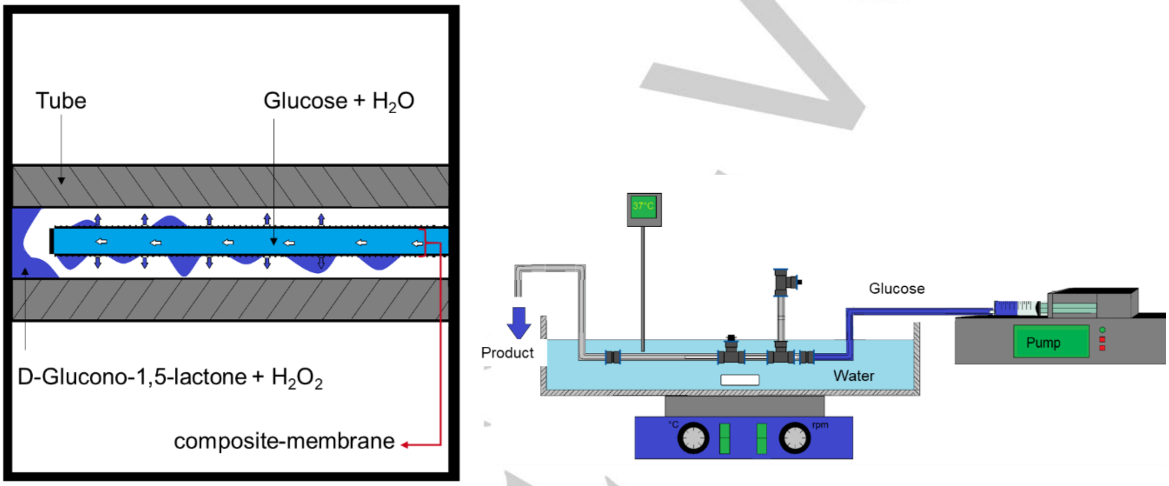

Figure S6. Schematic illustration of the experimental setup for the continuous enzymatic conversion of glucose in a GOD@ZIF-8 hollow fiber membrane reactor.

Fluorescent labeling of GOD with NHS-Fluorescein

For fluorescent enzyme labeling, NHS-Fluorescein was used. The labeling procedure was carried out according to the manufacturer's instructions. In a typical labeling experiment, 60 mg enzyme is dissolved in 1 mL PBS buffer solution (pH 7.4) and NHS-Fluorescein is dissolved in dimethyl sulfoxide (10 mg/mL). 266 µL of the NHS-Fluorescein solution is added to the enzyme solution and is incubated for one hour in a dark environment. After the labeling, the enzyme is purified by three centrifugation and washing cycles using Amicon®Ultra 0.5 mL centrifugal filters from Merk with an MWCO of 30 kDa.

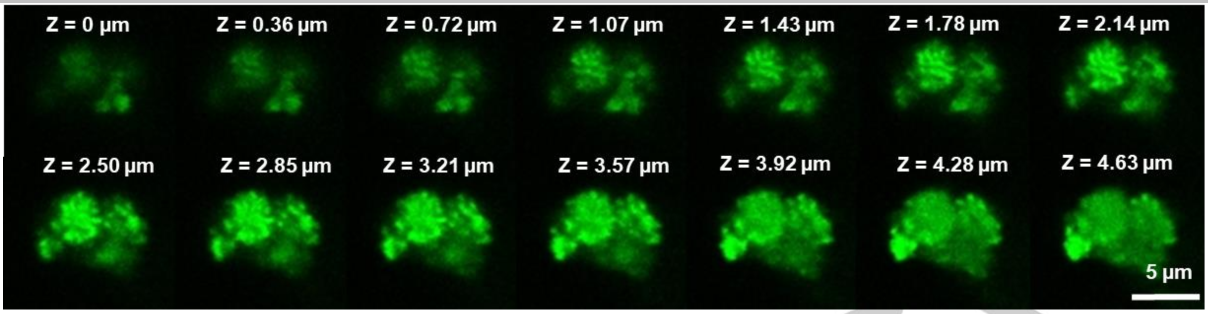

Figure S7. Confocal laser scanning microscope (CLSM) images of GOD@ZIF-8 with NHS-Fluorescein labeled GOD. Individual focal planes measured every 360 nm along the z-axis reveal a homogenous distribution of enzyme within the ZIF-8 crystals.

Glucose quantification

A colorimetric test was used for the quantitative measurement of glucose. The test is based on the reaction between dinitrosalicylic acid (DNSA) and reducing sugars described by Miller et al. For the analysis the DNSA solution is prepared by subsequently dissolving 1 g of 3,5-dinitrosalicily acid and 30 g of potassium sodium tartrate tetrahydrate in 50 mL ultrapure water followed by the addition of 20 mL 2 M sodium hydroxide solution and 30 ml ultrapure water. The colorimetric test was carried out by mixing 100 µL DNSA-solution with 100 µL glucose solution. After incubation at 80 °C for 10 min on a thermoshaker the samples are diluted with 1 mL ultrapure water. The sample is then analyzed in a plate reader at 525 nm.

Quantification of the immobilized enzyme mass in GOD@ZIF-8

The amount of immobilized GOD is quantified by bicinchoninic acid (BCA) assay as instructed by the manufacturer Alfa Aesar. Prior to the analyses, a coated membrane with a defined length is immersed into PBS buffer the pH is set to 3.5 by adding HCL, leading to a dissolution of the ZIF8 crystals. The samples are incubated for 24 h on a thermoshaker to ensure complete dissolution of the ZIF8 crystals. Before analysis, the enzyme is purified by three repetitive centrifugation and washing cycles using Amicon®Ultra 0.5 mL centrifugal filters from Merk with an MWCO of 30 kDa. The purified enzyme is resuspended in ultrapure water and stored at 4°C until further analysis.

$$encapsulation\ efficiency = \frac{c(GOD)_0 - c(GOD)_{after\ reaction}}{c(GOD)_0} \times 100$$

Equation S1

Optical analysis by electron and fluorescence microscopy

Electron micrographs were taken with a scanning electron microscope S4800 from Hitachi. The solid ZIF8 crystals are resuspended in ultrapure water and pipetted on a silicon wafer for analysis. The membranes are either broken in liquid nitrogen (cross-section) or cut with a scalpel (top view) in 5 mm pieces. All samples are dried overnight under vacuum and sputtered with 6 nm gold/palladium (80:20) before analysis. Fluorescence microscopy images are taken with an inverted laboratory microscope (Leica DM IL LED) or a Leica confocal laser scanning microscope (TCS SP8 FALCON).

X-ray diffraction

X-Ray Diffraction (XRD) analysis was performed using an Empyrean X-ray Diffractometer from PANalytical equipped with a Cu-anode. The analyses were performed with the following parameters: U = 40 kV, I = 40 mA, $\lambda K\alpha 1$ = 0.1540 nm, 5°- 45° at room temperature.

WILEY-VCH
